# Evaluation of High-Precision Sensors in Structural Monitoring

**DOI:** 10.3390/s101210803

**Published:** 2010-12-02

**Authors:** Bihter Erol

**Affiliations:** Geomatics Engineering Department, Civil Engineering Faculty, Istanbul Technical University, Maslak 34469 Istanbul, Turkey; E-Mail: bihter@itu.edu.tr; Tel.: +90-212-285-3821; Fax: +90-212-285-3821

**Keywords:** structural deformations, inclination sensor, GPS sensor, spectral analysis, engineering structures, geodetic monuments

## Abstract

One of the most intricate branches of metrology involves the monitoring of displacements and deformations of natural and anthropogenic structures under environmental forces, such as tidal or tectonic phenomena, or ground water level changes. Technological progress has changed the measurement process, and steadily increasing accuracy requirements have led to the continued development of new measuring instruments. The adoption of an appropriate measurement strategy, with proper instruments suited for the characteristics of the observed structure and its environmental conditions, is of high priority in the planning of deformation monitoring processes. This paper describes the use of precise digital inclination sensors in continuous monitoring of structural deformations. The topic is treated from two viewpoints: (*i*) evaluation of the performance of inclination sensors by comparing them to static and continuous GPS observations in deformation monitoring and (*ii*) providing a strategy for analyzing the structural deformations. The movements of two case study objects, a tall building and a geodetic monument in Istanbul, were separately monitored using dual-axes micro-radian precision inclination sensors (inclinometers) and GPS. The time series of continuous deformation observations were analyzed using the Least Squares Spectral Analysis Technique (LSSA). Overall, the inclinometers showed good performance for continuous monitoring of structural displacements, even at the sub-millimeter level. Static GPS observations remained insufficient for resolving the deformations to the sub-centimeter level due to the errors that affect GPS signals. With the accuracy advantage of inclination sensors, their use with GPS provides more detailed investigation of deformation phenomena. Using inclinometers and GPS is helpful to be able to identify the components of structural responses to the natural forces as static, quasi-static, or resonant.

## 1. Introduction

It is essential to determine the type, characteristics and scale of movements of a spatial structure or object under load from environmental forces to understand the potential probability of permanent damage or eventual destruction of the structure [1–6]. In metrology, various deformation monitoring and analysis approaches have traditionally been used [1,7–13] to ensure safe operation and usage of such structures. Furthermore, deformation may require very expensive restoration to be performed. The construction and management of such structures will be most cost-effective when the causes of deformation can be discovered and prevented. Currently, it remains an open question how best to detect significant deformations with an adequate level of precision [2,7,14–17].

In recent years, the progress of technology has resulted in improved precision of measurement systems, yielding more reliable results in deformation analysis of structures [18]. In addition to conventional terrestrial surveying methods using theodolites/total-stations, levels and similar surveying equipments, satellite-based positioning techniques (Global Navigation Satellite Systems, or GNSS, including GPS, GLONASS, Galileo, and Compass) have seen widespread use in structure monitoring. However, despite the many strengths and advantages of GNSS and conventional methods, there are still a few limitations due to the inherent geometric weaknesses of these systems, specifically, individual and atmospheric errors that affect their performance when used in high-precision work [1,14,17,19]. To overcome these techniques’ disadvantages and increase their level of accuracy, it is necessary to use additional precise measurement equipment, such as inclination sensors, accelerometers, and terrestrial laser scanners [1,6,7,12,13,15,20,21].

Today, inclination sensors are often part of measurement systems that are used in static and kinematic applications of many disciplines, such as measuring the inclination of land or platforms to build upon, in aircraft flight systems, vehicle security systems, volcano monitoring, precise farming *etc*. [22,23]. However, the studies are not many on the performance assessments of precise inclinometers and their use alongside other techniques for structural monitoring. In this study, a pair of inclination sensors of micro-radian precision were used alongside GPS for monitoring and determining the movements of a tall building and a geodetic monument (International GNSS Service-IGS continuous reference GPS station pillar), both located in Istanbul to the north of the North Anatolian Fault (NAF) [24–26]. Explaining merits and limitations of inclination sensors by comparing them to static and continuous GPS observations in deformation monitoring based on the drawn results of the case studies is purposed. The followed strategy and methodology for analyzing the deformations of case objects may constitute an example to the similar investigations.

At a glance, large differences can be seen in the specifications of commercially-available inclination sensors [22]. The inclinometers used in the study are dual axes digital sensors, able to determine simultaneously the degree and direction of inclination, along with the temperature. With an opto-electronic sensor unit, 0.001 mm/m measuring accuracy is achieved [27]. Since these sensors do not employ mechanically-driven moving parts, they do not need any maintenance and provide long-term stability, which are useful properties for continuous deformation monitoring.

In monitoring a tall reinforced concrete building which was damaged in the East Marmara Earthquake (M_w_ = 7.2) on 17 August 1999 [28], and then strengthened after the earthquake, two inclination sensors and dual-frequency geodetic GPS receivers were employed in the observation setup. Three static GPS campaigns were performed at six-month intervals. Considering GPS coordinate differences between the campaigns after datum transformation, the deformations were investigated to a millimeter-level (relative) accuracy [29]. Using the pair of inclination sensors, the structural movements of the building were continuously monitored for 36 days between the second and third GPS campaigns [30]. Through monitoring the deformations of the building, besides testing the performance of precise inclinometers versus GPS in deformation monitoring, we sought to analyze the long-term elastic deformations of the building in addition to record the probable static deformations that occurred under the effect of environmental forces, such as earthquakes and strong wind load.

In addition to the engineering structures, geodetic control network benchmarks are also subject to deformations under environmental effects and tectonic activities, and the distortions in a geodetic network affect the health and reliability of the geospatial infrastructure and cause crucial problems in every spatial data application. Therefore, we performed a second experiment with the inclinometers to monitor the movement of a geodetic monument, namely, a 1.20 m tall ISTA-IGS pillar. The pillar is located outdoors on the roof of a five-story reinforced concrete building on the campus of the Istanbul Technical University, and it carries a bullet type GPS antenna. Continuous monitoring of the pillar was required to explain the effects of movement from the host building, as well as atmospheric effects such as temperature, wind, and humidity. The inclination sensors continuously measured the movements of the pillar and the pier of the building, where the pillar is located, for two periods of approximately 30 days each in two different years, 2000 and 2002 [31,32]. Along with the observations from the inclination sensors, the baseline solutions were also obtained between the two continuous reference GPS stations, ISTA and KANT (a station of the Marmara GPS Network-MAGNET, which was established for geodynamic and earthquake research in the Marmara region) and the displacements of ISTA pillar was investigated depending on these solutions [33].

The time series obtained from the inclinometers and continuous GPS data were processed using the Least Squares Spectral Analysis (LSSA) technique, which is useful for the evaluation of periodic, multi-parameter, and noisy time series that can be decomposed into their component signals [34–37]. This method is based on the least squares approximation technique, which is closely related to least squares parametric adjustment [34,37], and it has certain advantages compared to classical Fourier methods, such as overcoming the blindness of former techniques to the existence of sampling irregularities or gaps in the data. It was applied in its original form by [38] and [39], and alternate forms have been used by a number of researchers in the fields of geodetic science [34,37,40,41–45], astronomy [46,47], geophysical applications (e.g., [48]), mathematics (e.g., [49]), microbiology (e.g., [50]), medicine (e.g., [51]), and finance.

In the overall conclusions from the case studies, besides the high accuracy advantage of inclination sensors in monitoring the dynamic structural movements at submillimetre level, detecting static or quasi-static deformations of the structure are not easy using these instruments. The displacements along the plumb line, like uplift or collapse of the structure, cannot be recognized using them. However, these limitations of inclination sensors can be defeated by GPS technique. Using static GPS observation method, the static or quasi-static deformations of a structure can be determined at the centimeter level. In literature, certain strategies, applying effective mitigation algorithms to GPS observations, are reported and suggested to improve the reliability of the GPS results [52–54]. In addition to its static applications, the GPS technique can be employed in continuous monitoring, and the analysis results of GPS time series and inclinations can be converted each other and hence provide a double check for continues structural movements. In the results of this study, although the inclination sensors were proved self-sufficiency in monitoring structural deformations, their use together with GPS sensors provide a more reliable results and detailed insight of structural behaviors. The results of this study should also be considered to emphasize the importance and necessity of structural health control using appropriate monitoring and analyzing techniques in active tectonic regions like İstanbul.

## 2. Description of the Observation Setup

### 2.1. Precise Inclination Sensors

Inclination sensors measure angular tilt with respect to an artificially-generated horizon (*i.e.*, a steady liquid surface), and they are used in a wide spectrum of applications, including land (or platform) leveling, aircraft flight controls, automobile security systems, and many civil engineering applications. Two specifications of these instruments, namely, precision and number of axes (which are usually, but not always, orthogonal), determine whether an inclination sensor is appropriate for a specific application [22].

**Figure 1.**
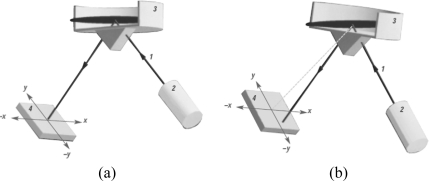
Functional principles of the inclinometer: **(a)** Horizontal position. **(b)** Tilted position. **1** The light from the LED, **2** LED, **3** Container with liquid inside, **4** Biaxial position detector [27].

In this study, two Leica NIVEL20 digital inclination sensors were employed [27]. These are precise dual-axis instruments with ±0.001 mm/m (±0.001 mrad) measuring accuracy within an effective range of ±1.5 mm/m and capable of measuring inclines and declines along the orthogonal directions (described as X and Y axes, intersect each other at an origin that defines a coordinate system). This axis definition is displayed on the sensor for the user’s reference, and thus, the directions of inclination can easily be identified (see Figure 1). The advantage of a dual-axis instrument is that it can observe the tilt of a surface and thus be able to interpret the movements of the observed object when it is deflected from the vertical.

In the design of the sensor, the horizon (tilt-sensitive element) is provided by a liquid in a closed container. The liquid’s surface is perpendicular to the vertical, independent of the sensor’s orientation. The inclination of the horizon relative to the sensor is measured by opto-electronic means. All the optical components used in the sensor are fastened to the underside of a flat glass plate. The plate also forms the bottom of the container with the transparent liquid. Through opto-electronic components, the luminous surface of a Light-Emitting Diode (LED) is imaged on a position-sensitive photodiode. The light from the LED is guided from below through the flat glass and the liquid, is totally reflected at the liquid’s surface, and then passes again through the liquid and the flat glass. Finally, the photodiode detects the position of the impinging light spot relative to the origin, which was adjusted and calibrated precisely in the horizontal configuration of the sensor (see Figure 1) [27].

**Figure 2.**
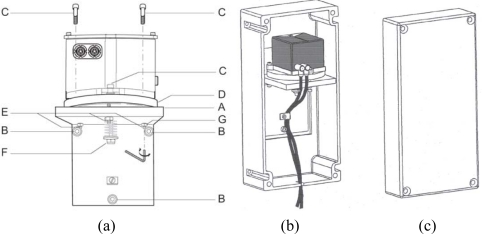
**(a)** Mounting elements of the NIVEL20 sensor. **(b)** The sensor on the support surface (steel console platform) with its plastic hood. **(c)** Cover of plastic hood [27]. A: Wall mount; B: 3 × M6 washer to install wall mount; C: 3 × M4 screws to install the sensor; D: Flange assembly to align the sensor in the desired direction by rotation; E: 3 × M5 ball pressure screws for coarse leveling of sensor; F: 1 × M6 center screw of the flange assembly; G: Nut to secure the leveling 3 × M5 ball pressure screws to ensure long-tem stability of the setup.

The flat glass plate, serving simultaneously as a component and as the support for the actual sensor element, is clamped onto a trough-shaped metal base. The sensor is set up on three hardened and ground circular support surfaces on the underside of this base. The support surfaces have through holes for the screws in the center. The sensor is covered with a plastic hood that serves primarily as a thermal insulator, which is meant to prevent external heat effects from causing non-uniform thermal expansion in the interior of the sensor and consequent measuring errors (see Figures 2(b) and (c)). A temperature sensor is also installed to monitor the sensor’s temperature. A bubble level on the sensor allows the operator a rough determination of the horizontal. More precise adjustment of the horizon is done electronically by the help of software before starting measurement with the instrument. The values of inclinations in the X and Y directions (in mrad, mm/m, or arc-min) and the temperature (in °C) are available as measurement values, and the observed data is visualized and stored using commercial software in a terminal computer. The inclinometer has an RS-232 or RS-485 serial interface, the latter of which has the capability of operating up to thirty-two sensors on the same network. Some specifications of the measuring capacity of the sensor are summarized in Table 1 [27].

**Figure 3.**
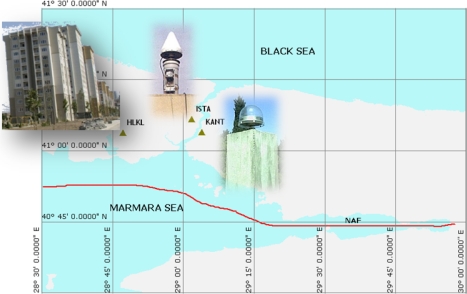
Locations of the tall building (HLKL) and the geodetic network stations (ISTA and KANT) in Istanbul. The red line indicates the approximate location of the North Anatolian Fault (NAF) (refer to [25] and [26] for active tectonic maps of Turkey).

Because the main objective of this research is to investigate the performance of inclination sensors in monitoring structural deformations, the sensors were deployed at the top (17th) floor of a 54-meter high building in Istanbul (see the location of the building *vs*. NAF in the Istanbul map in Figure 3), as the top floor is where the maximum displacements were expected.

**Figure 4.**
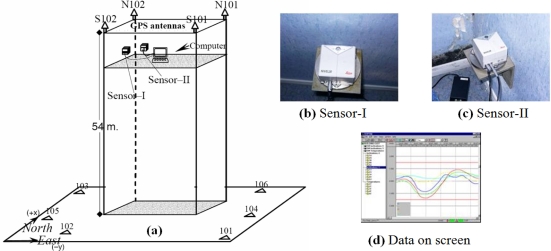
**(a)** Monitoring setup of the tall building: locations of inclination sensors and the deformation network benchmarks on and around the building [30], **(b,c)** instruments used in the study, **(d)** user interface of the monitoring software.

In the setup, each sensor was mounted on the piers of two orthogonal walls, and the orientation of the sensors on the steel mounting platforms (see Figure 4) was arranged so that the tilt components can be calculated in the employed reference system and can be compared to the other sensor. Precise adjustment of the sensors was done electronically before starting the measurements. For visualization and storage of the data, a laptop computer and an uninterruptable power supply (UPS) were placed next to the sensors, and software accompanying the inclinometers was used to manage the data (see user interface of the software in Figure 4(d)). The software interface allows the user to see incoming data either graphically or numerically during or after the observation period. Apart from a failure in the storage process at the beginning of the measurements, both instruments measured the inclination and temperature values during the whole campaign for 36 days at a sampling interval of about 5 minutes (irregularly occurred sudden changes in the data out of given sampling time is recorded automatically). The analytical results of the inclination and the results from the GPS processing were both used for deformation analysis of the building.

While the first experiment evaluates the indoor performance of inclinometers, the second test of the study clarifies their outdoor performance in deformation monitoring. In the second experiment, the structural deformation of the ISTA GPS reference station pillar was monitored. Since the pillar is on a building instead of a geologically safe location on hard terrain, and thus the self-movements of the building might affect its position, concerns about the stability of the pillar required its deformations to be investigated in the second phase of this study.

**Figure 5.**
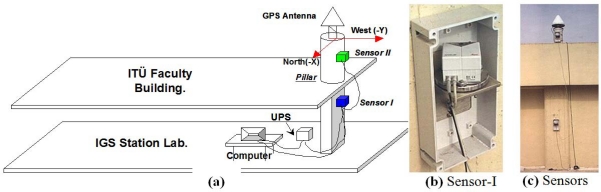
**(a)** Monitoring setup of the geodetic pillar [31], **(b,c)** Pictures from the campaign 2000.

The monitoring setup included the pair of inclination sensors, one of which was mounted on the pillar and the other on the building pier on which the pillar had been constructed (see Figure 5). The observations were performed in two individual campaigns between February and April in 2000 and 2002, with approximately 30 days of observation in each campaign. During the observation period, the sensors were protected against atmospheric effects like direct sunlight, rain, and snow. The sensors’ axes were oriented properly with the help of a compass to provide parallel reference systems. Thus, in both campaigns, the sensors’ X-axes were set approximately to the North-South direction and the Y-axes were in the East-West direction. They were connected to each other using an RS485 standard cable and then to a laptop computer using an RS232 standard cable. A UPS supplied power to the system to make it resilient to electricity outages (Figure 5). The inclination data and instant temperature values were collected at one-minute intervals and stored in a database for further analysis. Additionally, the daily weather conditions were also reported by an observer, providing valuable input for the interpretation of results.

### 2.2. GPS Observations

The satellite-based Global Positioning System (GPS) was originally introduced for navigation, but its high accuracy and reliability in positioning drove its adoption for structural deformation monitoring as well as other geodetic surveys, and subsequently for seismological studies [9,15,16,55,56]. With advantages in terms of accuracy and practicality, GPS proved its success for determining abrupt displacements and also very slow movements of structures, and this success motivated its application to studies of rapid displacements, for instance, recording the oscillations of bridges and other slender engineering structures [1,10,15].

There are, however, various measurement approaches for determining coordinates using geodetic GPS receivers, such as static (fixed GPS antenna in a certain position), kinematic (GPS antennas along a path), real-time kinematic (positioning in real time with receivers connected by radio or wire link), and post-processing kinematic. In each of these, the positioning principles remain the same: the GPS antenna represents the apex of an inverted pyramid, the base of which is usually defined by at least four satellites (with accurately-known coordinates), and information is continually received from the satellites during the entire observation session. Hence, the coordinates of the point (the receiver antenna) can be determined to average accuracy of a few millimeters after correcting for short-period errors that affect the GPS signals, such as atmospheric and tropospheric delays [10]. However, the accuracy and reliability of coordinates vary depending on certain factors that affect the quality of GPS measurements, namely, the satellite visibility, availability, and geometry; the quality of the transmitted signal and its delay by the ionosphere and troposphere; secondary reflections of the satellite signal by topographical objects (multipath signals); and personal errors like misreading the GPS antenna height or centering the antenna on a wrong benchmark [1,10,19]. To improve the positioning quality, therefore, special precautions and efforts may be required in GPS monitoring of deformations [52–54]. In any case, low accuracy is expected from observations taken in places where satellite visibility is poor, such as in canyons or streets surrounded by high buildings, as well as near reflecting surfaces where signals are noisy, such as water reservoirs and metal covers. The selection of receiver location and the duration of measurements, as well as software exclusion of low-quality measurements or signals (including “cut-off angles”, *i.e*., satellites at very low angles relative to the horizon and signals subject to strong atmospheric effects) permit us to control these sources of errors.

In addition to inclination sensors, GPS was also included in the monitoring setup of both experiments here. Hence, both techniques were compared, and their merits and limitations in deformation analysis were clarified. We can thus assess their individual and together contribution to the deformation monitoring. In each case study, a different GPS measurement and data analysis strategy was adopted.

In monitoring the tall building, three GPS campaigns were completed at six-month intervals, and the static GPS measurement method was applied. The control network for GPS monitoring, established at the beginning of the first campaign, can be seen in Figure 4. The benchmarks of the control network were located on stable hard terrain around the building (numbered 101 to 106), and four of the deformation benchmarks were also located at the tops of the piers of the building (S101, S102, N101, and N102) (Figure 4). Dual-frequency twelve-channel geodetic GPS receivers were used in the campaigns. The durations of the GPS sessions varied from 40 to 60 minutes, with 5-second sampling intervals. The GPS data of the campaigns were processed using default parameters of a commercial GPS data processing software (Leica Geo Office—LGO) and in the results the relative accuracy of GPS-derived positions of the deformation points is calculated better than ±3 millimeters.

In deformation analysis of the ISTA pillar using the GPS technique, the baseline solutions are considered between two continuous reference stations, namely, the ISTA and KANT pillars. The KANT geodetic pillar is located 6 km southeast of ISTA on geologically-stable hard ground (see the map in Figure 3). Both geodetic monuments are located on the same tectonic plate [24–26,33]. In the processing strategy, the coordinates of ISTA (Easting and Northing) were derived from the hourly GPS data of the stations with 30-second sampling intervals. The sequential solutions (24 solutions per day for approximately 30 days) of both measurement campaigns constituted the time series. Then, the periods of movement were determined using LSSA, and the results were compared with the output from the LSSA for inclination sensors data.

## 3. Methodology and Details of Data Analysis

### 3.1. Least Squares Spectral Analysis (LSSA) Method

Due to the nature of the measurements, the observed time series are assumed to be composed of two main constituents: signal and noise. The noise can be random and systematic. Uncorrelated random noise with constant spectral density, which is known as white noise, is assumed to be ideal. However, in practice, the observables mostly include non-white random noise, which is a band-limited random function of time. Systematic noise may also have systematic and non-systematic parts and can be modeled with certain mathematical forms. In contrast, non-systematic noise can include datum shifts and trends, which renders the series non-stationary. When analyzing the time series, a possible trend is generally identified and removed to avoid non-systematic noise [34,36], and de-trended data consists of superimposed signals.

Depending on the structure of the analyzed data, in some cases a particular signal of a time series can be dominant and can be modeled using simple techniques such as a parametric model or polynomial fitting. However, the measurements mostly have irregular distributions with unequal intervals, and in such cases spectral techniques are used to analyze the data as a sum of periodic signals and to estimate their periods and amplitudes. This can be done either using non-parametric methods, such as Fourier transform based algorithms, which are commonly applied to longer time series with equidistant data, or using parametric methods, such as Least Squares Spectral Analysis, a method to decompose a signal into a sum of trigonometric terms using least squares techniques, even for short time series with non-equidistant data.

Spectral Analysis by Least Squares was first developed and applied by Vani ek in 1969 [38,39] to overcome the inherent limitations of classical Fourier methods. Notable advantages provided by LSSA are: (*i*) the systematic noise (colored or otherwise) can be rigorously suppressed without causing any shift in the existing spectral peaks [40], (*ii*) time series with unequally-spaced values can also be analyzed without pre-processing [41], (*iii*) time series with an associated covariance matrix can be analyzed and (*iv*) statistical tests on the significance of spectral peaks can be performed, which makes the method promising and powerful for evaluating the time series.

In LSSA, an observed time series is considered to be a function of time *t_i_* and is represented by *f* = *f* (*t*) = {*f_i_*}, *i* = 1, 2,…,*n*. As the main objective of LSSA is to detect the periodic signals in *f*, especially when both random and systematic noise is present, *f* can be modeled with function *g* as follows:

(1) g=Φx

where Φ is a matrix of known base functions and *x* is the vector of unknown parameters. Here it is not necessary for the time series to be equally-spaced. However, the observations *f_i_* are assumed to possess a fully populated covariance matrix C*_f_*. To estimate the model parameters *x*, the standard least squares method (e.g., [51]) is used, in which the difference between *g* and *f* is minimized in the least squares sense. The estimate of model parameters can be obtained as follows:

(2) $$\hat{x}={\left(\Phi^{T}C_{f}^{-1}\Phi \right)}^{-1}\Phi^{T}C_{f}^{-1}f$$

(3) $$\hat{g}=\Phi \hat{x}=\Phi {\left(\Phi^{T}{\text{C}}_{f}^{-1}\Phi \right)}^{-1}\Phi^{T}{\text{C}}_{f}^{-1}f$$

In the least squares method, the model parameters are determined to minimize the difference between *ĝ* and *f*. Using the standard least squares [57], we obtain:

(4) $$\hat{v}=f-\hat{g}=f-\Phi {\left(\Phi^{T}{\text{C}}_{f}^{-1}\Phi \right)}^{-1}\Phi^{T}{\text{C}}_{f}^{-1}f$$

From the projection theorem, it is known that *v̂* ⊥ *ĝ*, which means that *f* has been decomposed into a signal *ĝ* and noise *v̂* (residual series). Thus, to describe how *ĝ* represents *f*, a fractional measure *s* that is the ratio of the length of this orthogonal projection to the length of *f* is used:

(5) $$s=\frac{f^{T}{\text{C}}_{f}^{-1}\hat{g}}{f^{T}{\text{C}}_{f}^{-1}f}$$

In spectral analysis, the hidden periodic signals that are expressed in terms of sine and cosine base functions are sought. Therefore, if a set of spectral frequencies (*ω_i_*, *i* = 1, 2,…,*m*) are specified, then we can express the signals as:

(6) $$\hat{g}(\omega_{i})={\hat{x}}_{1i}\text{cos}\omega_{i}t+{\hat{x}}_{2i}\text{sin}\omega_{i}t$$

Let *x̂* = [*x̂*_1*i*_, *x̂*_2*i*_]*^T^* and Φ = [cos *ω_i_t*, sin *ω_i_t*]. Then *x̂* can be determined from Equation (1). For different frequencies *ω_i_*, *i* = 1, 2,…,*m*, different spectral values can be obtained. Then, the least squares spectrum is defined by:

(7) $$s(\omega_{i})=\frac{f^{T}{\text{C}}_{f}^{-1}\hat{g}(\omega_{i})}{f^{T}{\text{C}}_{f}^{-1}f}={\left[1+\frac{Q_{n}}{Q_{s}}\right]}^{-1},\;\;\;\;\;i=1,\;2,\;\ldots ,\;m$$

where *Q_n_* and *Q_s_* are the quadratic norms of the noise and signal, respectively [34]. Equation (7) describes the least squares spectrum. Obviously, the least squares spectrum of *f* is the collection of the spectral values for all desired frequencies *ω_i_*, *i* = 1, 2,…,*m*. The greater the spectral value at a frequency *ω_i_*, the more powerful *f* is at this frequency [34]. Given Equation (7), statistically significant spectral peaks satisfy the following inequality:

(8) $$s(\omega_{i})\geq {\left[1+\frac{v}{2}F_{v,\;2,\;\alpha }\right]}^{-1}$$

It is obvious from Equation (8) that the least squares spectrum follows the Fisher distribution with *v* degree of freedom and *α* level of significance [34]. Also, Equation (7) can be further developed into other forms to provide the researcher with more familiar spectral representations, such as power spectral density (PSD) in decibels (dB) or in unit^2^/frequency, where ‘unit’ is the unit of the time series values [37]. In [34], the least squares PSD in decibels is given by:

(9) $${\text{PSD}}_{\text{LS}}=10\text{log}\left[\frac{s}{1-s}\right](\text{dB})$$

Solving Equation (7) with respect to *Q_s_* and dividing by the frequency *f*, the classical least squares spectrum can be transformed into the least squares PSD in unit^2^/*f*:

(10) $${\text{PSD}}_{\text{LS}}=\frac{Q_{n}}{f}\left[\frac{s}{1-s}\right]\left({\text{unit}}^{2}/f\right)$$

The least squares PSD in Equations (9) and (10) are equivalent to those determined from the Fast Fourier Transform (FFT) when the series is equally spaced and equally weighted. In addition, these equations can also be used to calculate the power spectra of any series, without the restrictive conditions of the FFT, which is the advantage of the LSSA method [37].

Generally speaking, the observed time series may include trigonometric base functions (Equation (6)) to describe the periodic components of the series, along with random walk and auto-regressive components. When the calculation of the least squares spectrum is carried out, there will be a simultaneous least squares solution for the parameters of the process. This approach is represented as a rigorous approach to the problem of hidden periodicities, where the parameters of the assumed linear system driven by noise are determined simultaneously with the amplitudes and phase of the periodic components, as well as with other parameters that describe systematic noise [34,37,58]. The LSSA software that implements the above algorithm was used in the spectral analyses of data in this study [34,36].

### 3.2. Case Study-I: Analyzing Movements of the Tall Building

**Figure 6.**
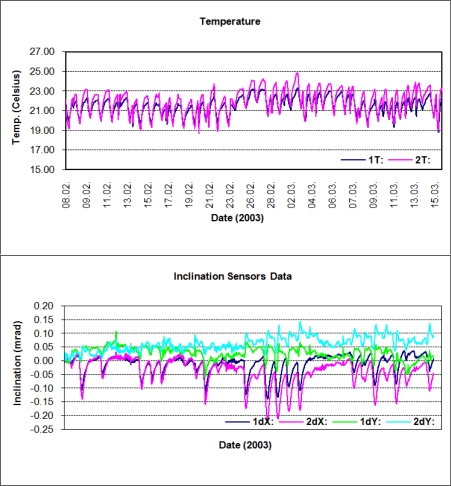
Temperature changes and inclinations of *the tall building* from 8 February to 15 March 2003.

In this section, we discuss the results from the inclination sensors and GPS data for clarifying the behavior of the building. First, we present the raw temperature and inclination data from the sensors. In Figure 6, the first chart shows the indoor temperatures recorded by the sensors, varying between 19 °C and 24 °C. The outdoor temperatures over the observation period ranged between 4 °C and 11 °C. The second chart shows the inclination data in milliradians (mrad) along the Y (east-west) and X (north-south) axes. In the legends, the data are labeled dX and dY, since these values are relative to an initial value of tilt, which was set to approximately zero at the beginning of the observations. In Table 2, the ‘magnitude’ corresponds to the maximum displacement of the observed object [30].

The LSSA of the inclination data was performed in two steps. In the first step, all observations shown in Figure 6 were taken into account and analyzed. In the second step, involving more rigorous investigation of the spectrum, the data was divided into weeks and LSSA was applied to weekly data. To expose higher frequencies in the data, the significant signal with a 24-hour period was suppressed. In the end, as expected, the LSSA of Sensor-I and Sensor-II data revealed identical results, and therefore only the results from Sensor-I data are shown here.

**Figure 7.**
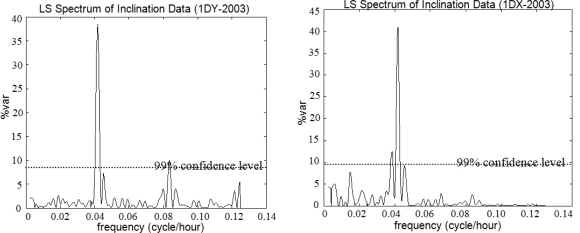
Least Squares (LS) spectrum of *the entire observations*.

**Figure 8.**
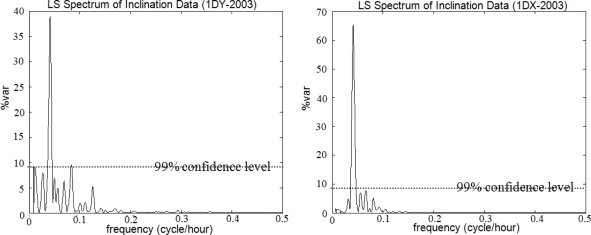
LS spectrum of *weekly observations*.

The outputs were as follows: the periods, frequencies (cycle/hour), amplitudes with root mean square errors (RMSE), percentage variance levels (%var, which is a ratio showing how much of the signal *ĝ* is contained in the observed time series *f* from Equation (5)), and respective PSDs of the periodic signals. Significance tests were performed on the parameters. The variance levels, as derived in the first step, are plotted against frequency in Figure 7, where the significance of the signal with a 24-hour period (T = 24 h) is seen in both the Y and X lines. Nearly 40% of the signal with T = 24 h can be recognized in the observed data with LSSA. This outcome is supported by the evaluation results of the weekly observations (see Figure 8).

**Figure 9.**
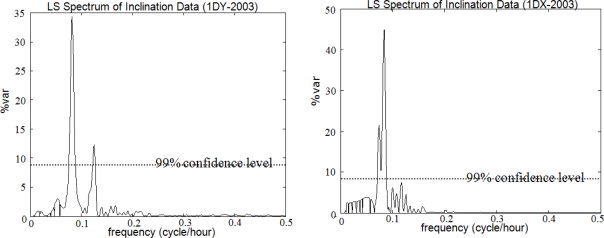
LS spectrum of *the observations after the signal with the highest period* is removed.

After the signal with T = 24 h, which was marked as significant in the previous steps, was suppressed, the significant peak in the X-axis data (inclinations on the north-south direction) with T = 12 h was recognized. In the observations on the Y-axis (east-west direction), an additional signal with T = 8 h was also identified. The significance of the higher frequencies in the Least Squares spectrum is clearly visible in Figure 9. The results of the evaluations are summarized in Table 3.

After analyzing the building movements in the frequency domain based on inclination data, we evaluated them in the space domain using GPS data [30]. The data from three GPS campaigns conducted in September 2002, February 2003, and August 2003 were processed and hence three coordinate sets were calculated for the deformation points after the adjustment. The RMSE of the coordinates was around ±3 millimeters, and three times the RMSE was adopted as the significance level with 99% confidence. In the assessments, the coordinate differences among the sets were also considered.

**Figure 10.**
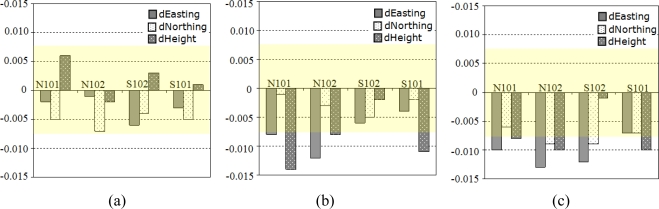
Coordinate differences of **(a)** the 1st and 2nd campaigns **(b)** the 2nd and 3rd campaigns **(c)** the 1st and 3rd campaigns (in meters) at the deformation benchmarks. Confidence intervals at 99% confidence level are indicated with a transparent layer on the graphs.

Easting, northing, and height differences of the N102, N101, S102, and S101 deformation points are shown in Figure 10. In the graphs, no significant movement can be seen between the first and the second campaigns. However, between the second and the third campaigns, significant changes of less than 1.5 cm are recorded in the easting of N101 and in the heights of N102 and S101. In the time between the first and third campaigns, significant changes are seen in the easting components of N101, N102, and S102. The only change in the easting of N102 is less than 1 cm. In the heights of N101 and S101, a 1-cm change is also found to be significant.

From the GPS data analysis, although the significant displacements of the building seemed to be proven, these results do not give an idea to distinguish the components of the movement as static, quasi-static or resonant.

### 3.3. Case Study II: Analyzing Movements of the Geodetic Pillar

**Figure 11.**
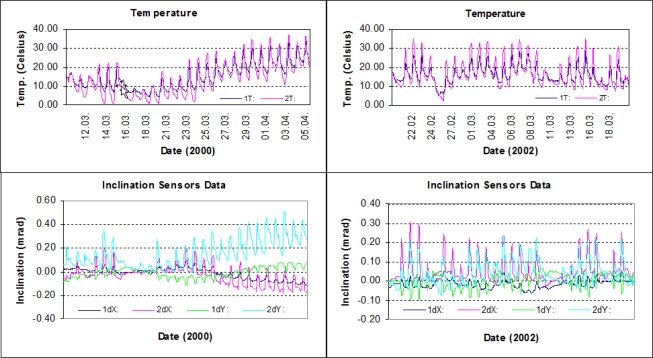
Temperature and inclination data of *the geodetic pillar*. Campaigns ran from 10 March to 6 April 2000 and from 20 February to 22 March 2002 [32].

In investigating the movements of the ISTA geodetic pillar, an analysis procedure was followed that was similar to the one used for the tall building. Inclination data from two years’ campaigns, along with the GPS baseline solutions of each campaign, were analyzed independently using the LSSA method. In the main conclusions of the inclination data evaluations, it was seen that the pillar moved similarly in the 2000 and 2002 periods, and the results from the GPS data analysis supported this conclusion. For a better understanding of the results, we took a closer look at the data and the conditions during the observations. The temperature (in °C) and the inclination data (in mrad) of the sensor pair are illustrated in Figure 11, and the main statistics of the inclination data are presented in Table 4.

According to the corresponding temperature graphs and the observer reports, the weather conditions seemed to be generally stable during both campaigns. In observations in the year 2000, the temperature varied between 5 °C and 32 °C, with a 2 °C difference between the two sensors on any given day and increasing to 7 °C at nights. After the 14th observation day, the average daily temperature increased, and this appeared to also affect the inclinations of the pillar (see the trend of inclination data, especially of the 2nd sensor, after the 14th day of observations in Figure 11). In 2002, the temperatures varied between 3 °C and 35 °C. The daily averages appeared to be consistent throughout the campaign. However, it should be noted that the temperatures measured are the values inside the plastic hoods.

**Figure 12.**
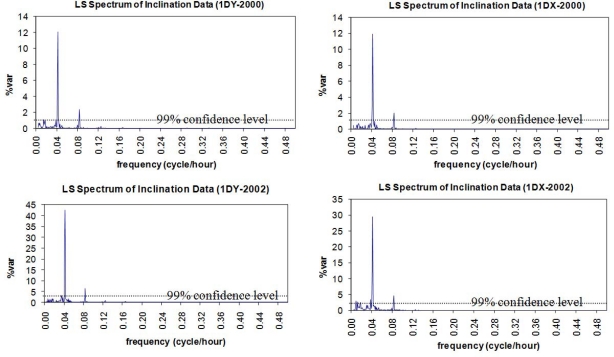
LS spectrum of inclination data of *Sensor I mounted on the building* that hosts the pillar. Campaign 2000-Y axis (1DY-2000) and X axis (1DX-2000). Campaign 2002-Y axis (1DY-2002) and X axis (1DX-2002).

**Figure 13.**
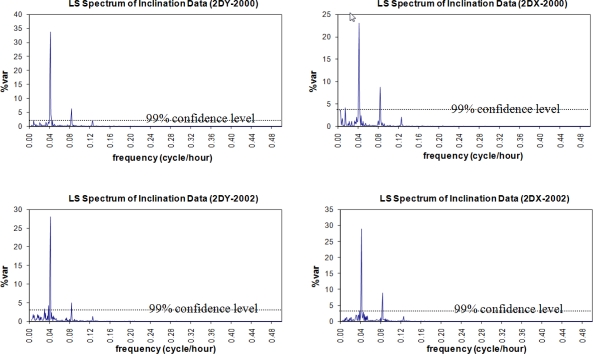
LS spectrum of inclination data of *Sensor II mounted on the pillar*. Campaign 2000-axis Y (2DY-2000) and axis X (2DX-2000). Campaign 2002-axis Y (2DY-2002) and axis X (2DX-2002).

In spectral analysis results of the inclinations, the hidden periodicities of the movements of the pillar and the building that hosts the pillar were clarified. The data analysis for the year 2000 yields the fact that both the pillar and the building seemed to have similar characters of movement as they did in 2002. In the overall results, the significant peaks with T = 12 h and T = 24 h were identified in the signals by Sensors I and II along the north-south (X-axis) and east-west (Y-axis) directions. The graphs in Figures 12 and 13 verify these conclusions.

Table 5 shows the amplitudes of the periodicities. When the periods of the significant peaks are considered, we can recognize the similarity in the resonant movements of the pillar (observed by Sensor-II) and the building (observed by Sensor-I, Figure 5). However, the amplitudes of the periodic signals show that the displacements of the pillar are greater than those of the building. In the 2002 results, the amplitude of the signal with T = 12 h on the X-axis is 5 microradians for the building and 25 microradians for the pillar). The analysis results of both campaigns were proved against each other.

**Figure 14.**
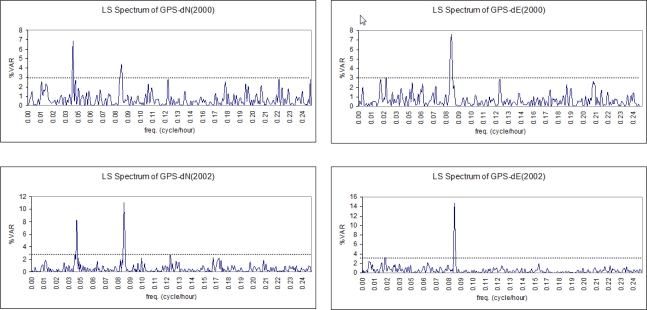
LS spectrum of GPS data (time series of the northing (dN) and easting (dE) components of the *ISTA pillar position*) for both campaigns in 2000 and 2002.

Inspecting the deflections of the pillar using the hourly data of continuous GPS reference stations generally supported the results of the previous findings. In the variance *vs*. frequency graphs in Figure 14, we can clearly see the significant peaks derived from the time series of the GPS solutions. Where the signal for the northing component (dN) contains periods with T = 24 h and T = 12 h, the easting component (dE) includes only the period with T = 12 h for both campaigns. In the results, the amplitudes of the significant peaks of the signal contents are around 0.5 mm.

### 3.4. Results and Discussion

The results from the case studies are as follows:

- LSSA of the inclination data showed that the tall building (HLKL) has significant periodic movements with 24-h and 12-h periods along the north-south and east-west directions. Another periodic movement with an 8-h period was identified along the east-west direction. As expected, the data from Sensor-I and Sensor-II, mounted on the inner faces of the west and north walls, respectively, showed similar results.
- Since 1 mrad = 0.001 mm/m and the height of the building is 54 meters, we can derive the horizontal displacements of the building in mm. Thus, the movement with T = 24 h has 0.024 mrad and 0.013 mrad amplitudes, corresponding to 1.3 mm and 0.7 mm horizontal displacements, respectively, in the north-south and the east-west directions. These amplitudes of displacement are judged to be safe for the building.
- In the GPS analysis results, no significant displacement is captured between the first and the second campaigns. However, in the time between *the second and the third campaigns*, in which inclination observations were also performed between these campaigns, a significant change of 1.3 cm was detected in Easting of N101. Additionally, there are significant displacements of 1.1 cm and 1.4 cm in the heights of S101 and N102, respectively.
- When the differences in the GPS coordinates between *the first and the third campaigns* were investigated, significant movements were seen at the centimeter level for every deformation point. In the easting of N102, N101, and S102, the differences are 1.0 cm, 1.3 cm, and 1.2 cm, respectively. In the vertical direction, N101 and S101 moved by 1.0 cm.
- In the results of GPS analysis, although the displacements were found to be significant at the chosen confidence level, previous experience with this technique has shown that it would not be realistic to determine the displacements to less than a centimeter. Considering the precision of geodetic GPS, the results obtained can be said to be too optimistic [15,19]. The horizontal displacements revealed from the inclination data analysis also support this supposition.
- When the movements of the ISTA geodetic pillar and its host building were investigated using inclination data from the two campaigns, periodic movement was found in both structures with T = 12 h and T = 24 h.
- The horizontal displacements of the host building are 0.30 mm/0.20 mm in 2000 and 0.40 mm/0.20 mm in 2002 along the east-west and north-south directions, respectively.
- The horizontal displacements of the pillar are 1.07 mm/0.67 mm in 2000 and 0.71 mm/0.79 mm in 2002 along the east-west and north-south directions, respectively.
- The LSSA results on GPS data are consistent with the inclination sensor evaluations. Movements with T = 24 h and T = 12 h to the north and with T = 12 h to the east were confirmed. The 0.50 mm displacement supports the previous findings as well.
- Considering that ISTA is a member of a global geodetic network and its position data is broadcasted for used as a reference for precise positioning in geodetic and geophysical applications, the amount of displacement is considered to be safe and can be ignored. Thus, no precaution appears to be required to improve its steadiness.

Overall, we can draw the following conclusions :

- The repeating T = 24 h movements of the tall building (HLKL), ISTA geodetic pillar, and its host building along the east-west and north-south directions can be interpreted as the natural responses of these structures to the warming and cooling phenomena in a day.
- However, the harmonics with a period shorter than 24 hours may nevertheless be the consequence of daily periodicity as a result of non-linear response of the structure. The environmental effects under consideration should not have fundamental periods of 12 h and 8 h.
- In terms of accuracy, it is evident from the results of the first case study that static observations with the GPS technique remained insufficient to resolve the deformations observed by the inclination sensors because of the error sources that affect GPS observations [19]. However, from the application point of view, using inclination sensors and GPS together in structural monitoring and analysis yields improved insight into deformation phenomena. To identify the response of the structure to environmental loading as static, quasi-static or resonant, the separate contributions of GPS and inclination sensors are both notable. In other words, the static application of GPS helps to identify the static or quasi-static deformations of a structure at the centimeter level. On the other hand, using inclination sensors provides input for clarifying the amplitude and frequency of periodic movements, even at the sub-millimeter level.
- In addition to its static application, the GPS technique can be used for continuous monitoring. In the second case study, the structural deformations of the pillar were analyzed using time series by continuous GPS solutions. The periodic components of the deformation signal were similarly clarified in inclination data evaluation. The daily movement of the structure is clear from the spectral analysis results of the GPS data.
- The other important output of this study is methodological. We confirmed and demonstrated the previously mentioned advantages of the LSSA technique in the spectral analysis of irregularly sampled data. Practical data handling and analysis strategy of the technique provided benefits in the evaluation of data and the interpretation of the results.

## 4. Conclusions

It is essential to analyze the behavior of structures under static/dynamic loading and to clarify their response to these forces to ensure their safe operation and use as well as to prevent the occurrence of deformations. Structural analysis is of even higher priority if the region is subject to natural hazards. Istanbul, one of the most crowded metropolises of the world, is located close to an active fault, has experienced two recent devastating earthquakes, and, according to many studies, is anticipating another strong earthquake (>7 Mw) within 30 years [5,59]. Aware of the risk to the city, many governmental offices, universities, and civil organizations have dedicated their efforts toward reducing the expected loss from the forthcoming disaster, and the data and suggestions based on accurate *in situ* experiments provide valuable input for these efforts.

From this point of view, this study was carried out in Istanbul to monitor and analyze self-movements of a reinforced concrete building and a geodetic pillar established on a building in a post-seismic period, using precise GPS and inclination sensors. We were able to determine the benefits of using inclinometers in structural monitoring itself and of obtaining support from static and continuous mode GPS observations from the geodetic point of view. In addition, we suggested a useable strategy for analyzing the structural behaviors. The results obtained were therefore satisfying.

In the results, the performance of precise dual-axis inclination sensors is sufficient for continuous monitoring and for determining the dynamic structural movements at a submillimeter level. However, it is not possible to use the sensors to derive static or quasi-static components of the motion. The other limitation in measuring with these instruments is that the displacements in the Z direction (vertical) cannot be examined directly, and therefore, only the displacements along the X and Y directions can be subjected to analysis. On the other hand, precise GPS observations made full-scale investigation of static and quasi-static structural responses possible in three dimensions. Therefore, inclination sensors and the GPS technique are complementary to each other.

The noise in the content of GPS signals (as seen in LS spectrums in Figure 14) decreases the data quality in the low-frequency range and hence its reliability for detecting the structural displacements. To overcome these disadvantages and obtain reliable results, appropriate strategies must be carefully applied in the form of monitoring and effective mitigation algorithms and procedures in data processing. Even when the proposed precautions are applied, it is also beneficial to double-check through the integration of the inclination and GPS sensors. Since the measurements convert quite well to each other, it is not difficult to directly compare the outputs of the two techniques.

In addition, due to the ongoing modernization of GPS satellites with additional navigation signals [60,61], along with expected improvements in GNSS from the ability to integrate signals from additional satellite constellations, GNSS shows great promise in terms of accuracy and reliability for future investigations of structural deformations.

## Figures and Tables

**Table 1.** Specifications of the inclinometer [27].

**Specification**	**Value**
measuring range	±1.5 mrad (or mm/m)
accuracy	±0.001 mrad
resolution	0.001 mrad
zero-point stability	<0.005 mrad/°C
operating temperature range	−20 to +50 °C
storage temperature range	−30 to +60 °C
relative humidity	10 to 95%
dimensions of the instrument (L × W × H)	90 × 90 × 63 mm
weight of the instrument	850 g
supply voltage	12 V DC ±25%

**Table 2.** Statistics of *the tall building* inclinations in mrad.

**Sensor No\Axis**	**Sensor-I\Y**	**Sensor-II\Y**	**Sensor-I\X**	**Sensor-II\X**
**maximum**	0.107	0.144	0.036	0.031
**minimum**	−0.059	−0.001	−0.141	−0.221
**magnitude**	0.166	0.145	0.177	0.252

**Table 3.** LSSA results of inclination data: periodicities of resonant movements of *the tall building*.

**Period (hour)**	**Direction**	**Frequency (cyc/hour)**	**Amplitude (mrad)**	**Direction**	**Frequency (cyc/hour)**	**Amplitude (mrad)**
**8**	Y-axis (east-west)	0.125	0.007	X-axis (north-south)	–	–
**12**		0.083	0.012		0.083	0.012
**24**		0.042	0.013		0.042	0.024

**Table 4.** Statistics of 2000 and 2002 campaigns: inclinations in mrad, *ISTA geodetic pillar*.

**Year**	**2000**				**2002**			

**Sensor No\Axis**	**I\Y**	**II\Y**	**I\X**	**II\X**	**I\Y**	**II\Y**	**I\X**	**II\X**
**maximum**	0.080	0.512	0.093	0.201	0.072	0.233	0.052	0.305
**minimum**	−0.114	−0.013	−0.114	−0.182	−0.100	−0.077	−0.065	−0.026
**magnitude**	0.194	0.525	0.207	0.383	0.172	0.310	0.117	0.331

**Table 5.** Overall LSSA results of inclination data: *geodetic pillar* (S-II) and *host building* (S-I).

		**Year 2000 Campaign (10 March to 6 April)**			
		**Y-axis (east-west)**		**X-axis (north-south)**	
	**Period (hour)**	**Frequency (cyc/hour)**	**Amplitude (mrad)**	**Frequency (cyc/hour)**	**Amplitude (mrad)**
**SENSOR-I**	**12**	0.083	0.008	0.083	0.006
	**24**	0.042	0.018	0.042	0.015
**SENSOR-II**	**12**	0.083	0.031	0.083	0.026
	**24**	0.042	0.062	0.042	0.039

		**Year 2002 Campaign (20 February to 22 March)**			
		**Y-axis (east-west)**		**X-axis (north-south)**	
	**Period (hour)**	**Frequency (cyc/hour)**	**Amplitude (mrad)**	**Frequency (cyc/hour)**	**Amplitude (mrad)**
**SENSOR-I**	**12**	0.083	0.010	0.083	0.005
	**24**	0.042	0.026	0.042	0.013
**SENSOR-II**	**12**	0.083	0.016	0.083	0.026
	**24**	0.042	0.041	0.042	0.046
